# Annexin A5 regulates hepatocarcinoma malignancy via CRKI/II-DOCK180-RAC1 integrin and MEK-ERK pathways

**DOI:** 10.1038/s41419-018-0685-8

**Published:** 2018-05-25

**Authors:** Xujuan Sun, Shuqing Liu, Jinxia Wang, Bin Wei, Chunmei Guo, Chen Chen, Ming-Zhong Sun

**Affiliations:** 1 Department of Biotechnology, Dalian Medical University, 9 West Section, Lvshun Southern Road, Dalian, 116044 China; 2 Department of Biochemistry and Molecular Biology, Dalian Medical University, 9 West Section, Lvshun Southern Road, Dalian, 116044 China

## Abstract

As a calcium-dependent phospholipid binding annexin protein, annexin A5 (Anxa5) links to the progression, metastasis, survival, and prognosis of a variety of cancers. Current work showed ANXA5 overexpression was positively correlated with the upregulations of CRKI/II and RAC1 in hepatocarcinoma (HCC) patients’ tissues, which potentially enhanced the clinical progression and lymphatic metastasis of HCC. The role and action mechanism of ANXA5 in hepatocarcinoma was then investigated using a hepatocarcinoma Hca-P cell line, an ideal and well-established murine cell model with 100% inducible tumorigenicity of implanted mice with low (~25%) lymph node metastatic (LNM) rate. In vitro evidences indicated ANXA5 stable knockdown resulted in decreased proliferation, migration, invasion and adhesion to lymph node (LN), and increased intercellular cohesion behaviors of hepatocarcinoma Hca-P cells. Consistently, stable ANXA5 knockdown led to reduced in vivo tumorigenicity and malignancy, LNM rate and level potentials of Hca-P- transplanted mice via inhibiting CD34 and VEGF3. The levels of CRKI/II and RAC1 were reduced in tumor tissues from mice transplanted with Hca-P cells with stable ANXA5 knockdown. Molecular action investigation further showed ANXA5 downregulation apparently suppressed the expressions of molecules CRKI/II, DOCK180, RAC1 in integrin pathway, p-MEK, p-ERK, c-Myc, and MMP-9 in MEK- ERK pathway together with VIMINTIN in Hca-P cells in appropriate to knockdown extent. Collectively, Anxa5 was able to mediate HCC carcinogenesis via integrin and MEK-ERK pathways. It is of potential use in the research and treatment of HCC.

## Introduction

Listed as 5th and ranked as 3rd highest mortality among the most common cancers, hepatocarcinoma is an increasing health problem worldwide with high occurrence, metastasis and poor prognosis^1,2^. Generally, the early malignancy of lymphatic metastasis is clinically considered as a major prognostic indicator of cancers^3,4^. The lymph node metastasis (LNM) leads to a decrease of 50% for the prognosis of cancer patients. The study on the metastasis mechanism, specifically on the lymphatic metastasis, benefits better understanding of the diagnosis, prognosis and treatment of hepatocarcinoma.

Anxa5 is a member of the group A of annexin family that are Ca^2+^-regulated phospholipid-binding proteins with 12 members, annexin A1–A11 and A13. Commonly activated by higher cellular concentration of Ca^2+^ or higher content of phosphatidylserine (PS), monomeric ANXA5 spontaneously forms trimer through binding to the cell membrane for exhibiting its functions^5,6^ in physiological and pathological processes such as cell differentiation, apoptosis, signal transduction, inflammation and coagulation^7–10^. The dysexpression of Anxa5 is associated with the development, progression, metastasis, treatment and prognosis of a variety of cancers^11–14^. As we summarized the researches on Anxa5 in carcinogenesis^15^, Anxa5 expression level were commonly correlated with increased progression, metastasis, poorer survival and prognosis of most types of cancers. However, the underlying molecular regulation mechanism and clinical significance of Anxa5 in cancer progression and metastasis, especially in cancer lymphatic progression and metastasis, are poorly understood.

Research work from our group established the positive correlation of Anxa5 expression level with hepatocarcinoma progression and lymphatic metastasis^16–19^. Higher expression level enhanced the in vitro and in vivo tumor malignant progression and LNM potential of Hca-P, a murine hepatocarcinoma cell line with 100% tumorigenicity and ~25% LNM rate being proved as an ideal model for mimicking the initial lymphatic metastasis of hepatocarcinoma cell and hepatocarcinoma progression in clinical^20–24^. In current work, we found ANXA5 was overexpressed in tumorous tissues from HCC patients and correlated with CRKI/II and RAC1. Cellular experimental results showed that stable knockdown of ANXA5 led to decreased in vitro migration and invasion capacities as well as clearly reduced in vivo tumorigenicity velocity and malignancy, LNM rate and level potentials of Hca-P- transplanted mice via inhibiting the expressions of CD34, VEGF3, CRKI/II, and RAC1. Current work also revealed the critical molecules CRKI, CRKII, DOCK180, and RAC1 in integrin pathway, as well as p-MEK, p-ERK, c-Myc, and MMP-9 in MEK-ERK pathway were liked to ANXA5 dysexpression in Hca-P cells. Anxa5 expression mediates the progression and metastasis of HCC via integrin and MEK- ERK pathways. It is of potential use in HCC diagnosis and treatment.

## Results

### ANXA5 overexpression correlates with HCC progression and metastasis

**Fig. 1:**
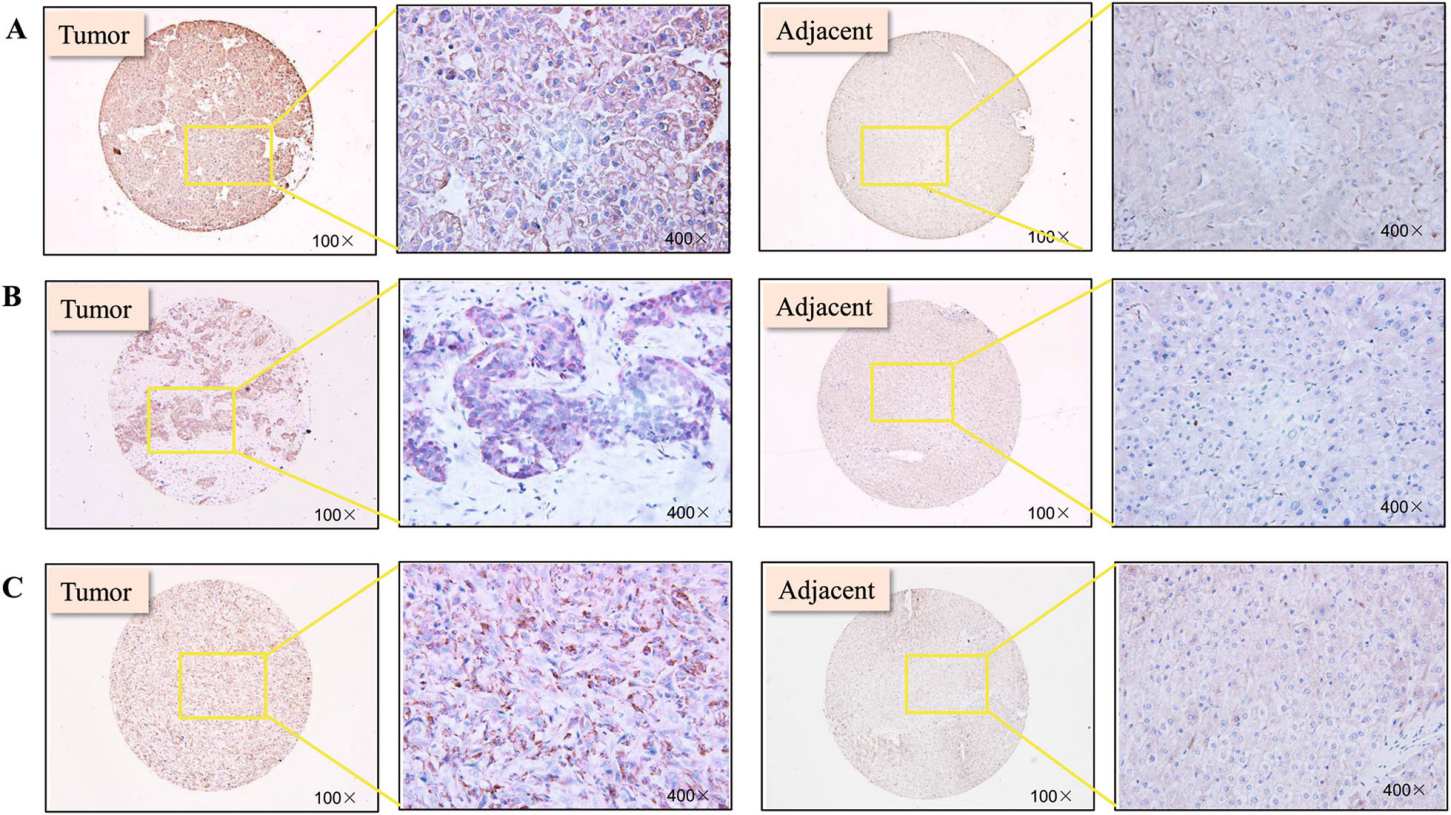
ANXA5, RAC1, and CRKL expression profiles and correlationships in tumors tissues of HCC patients. **a** ANXA5, **b** CRKI/II, and **c** RAC1 were overexpressed in tumors tissues from 46 HCC patients (Table 1). ANXA5 (*P* = 0.015) and RAC1 (*P* = 0.015) overexpression was related to the TNM stage (Table 2). CRKI/II was positively correlated with the gender of HCC patients (*P* = 0.029). The positive correlations of the expression levels of ANXA5 with CRKI/II (*P* = 0.009, *R* = 0.512) and RAC1 (*P* = 0.0003, *R* = 0.761), and RAC1 with CRKI/II (*P* = 0.001, *R* = 0.465) (Table 3)

A tissue microarray composed of 46 paired tumorous and adjacent normal tissues from HCC patients was employed to address the expression alteration of ANXA5. As shown in Fig. 1a and Table 1, ANXA5 was upregulated in patients’ tumorous tissues. The change was analyzed for its relevance with the clinicopathologic parameters of HCC patients. As shown in Table 2, its dysexpression was related to TNM stage (*P* = 0.015). Among 46 HCC patients, 3 patients diagnosed with LNM all showed immunoreactivities against ANXA5 with the highest degree +++ (*P*= 0.014). The results implicated ANXA5 level was positively correlated with the progression and metastasis of HCC.

**Table 1:** ANXA5, CRKI/II and RAC1 was overexpressed in tumor tissues from 46 HCC patients

Protein	Group	Total	−	+	++	+++	*P*
ANXA5	Tumor tissues	46	7	12	18	9	0.001
	Adjacent tissues	46	31	11	3	1	
CRKI/II	Tumor tissues	46	3	7	33	3	0.002
	Adjacent tissues	46	5	22	17	2	
RAC1	Tumor tissues	46	1	1	25	19	0.021
	Adjacent tissues	46	3	5	26	12

**Table 2:** ANXA5, CRKI/II and RAC1 overexpression was correlated with HCC progression and metastasis

Parameters	**Total**	**ANXA5**				***P***	**CRKI/II**				***P***	**RAC1**				***P***
		**−**	+	++	+++		**−**	**+**	**++**	**+++**		**−**	**+**	**++**	**+++**	
Age, years						0.219					0.795					0.261
< 60	37	6	11	13	7		3	5	27	2		1	1	21	14	
≥ 60	9	1	1	5	2		0	2	6	1		0	0	4	5	
Gender						0.499					0.029					0.482
Male	35	5	9	15	6		2	2	29	2		1	0	17	17	
Female	11	2	3	3	3		1	5	4	1		0	1	8	2	
Edmondson grade						0.219					0.165					0.889
I–II	22	1	8	9	4		3	4	14	1		1	0	11	10	
III–IV	19	6	4	6	3		0	3	14	2		0	0	10	9	
TNM stage						0.015					0.117					0.011
I–II	23	4	10	7	2		3	4	15	1		1	0	17	5	
III–IV	23	3	2	11	7		0	3	18	2		0	1	8	14	
AFU, U/mL						0.814					0.991					0.345
≤20	17	0	6	9	2		1	2	14	0		1	0	11	5	
20–400	9	3	0	3	3		1	1	6	1		0	0	4	5	
>400	19	3	6	6	4		1	4	12	2		0	1	10	8	
HBsAg						0.38					0.479					0.420
Negative	9	2	4	1	2		0	3	6	0		0	0	7	2	
Positive	33	5	8	14	6		3	4	23	3		1	0	18	14	
HBsAb						0.251					0.663					0.53
Negative	37	7	9	14	7		3	6	25	3		1	0	24	12	
Positive	3	0	3	0	0		0	0	3	0		0	0	1	2	
HBeAb						0.333					0.794					0.472
Negative	8	2	1	3	2		0	2	5	1		0	1	5	2	
Positive	33	4	11	13	5		3	5	23	2		1	0	20	12	
HBcAb						0.261					0.622					0.308
Negative	5	1	0	2	2		0	1	4	0		0	0	2	3	
Positive	37	6	12	13	6		3	6	25	3		1	1	23	12	

### CRKI/II and RAC1 are unregulated and synergistically correlated with ANXA5 in HCC progression

We also measured the relative expression levels of CRKI/II and RAC1 in above tissue array of HCC patients. Compared with the adjacent normal tissues, CRKI/II was upregulated in HCC tumorous tissues (Table 1, *P* = 0.002). We analyzed the CRKI/II deregulation with the clinicopathologic parameters of patients. As shown in Table 2, CRKI/II overexpression was not related to the age, Edmondson grade, AFU or HBsAg level ( *P*> 0.05), but interestingly, was significantly correlated with gender ( *P*= 0.029). The ratio of male patients (89%) with CRKI/II ++ and +++ expression level was ~2 times of that in female (45%). CRKI/II showed the trend more positively and higher expressed in patient tissues with higher TNM stages (Table 2). The result suggests the clinical possibility of ANXA5 mediating HCC malignancy via CRKI/II. RAC1 was also detected up-regulated in the tumorous tissues than adjacent normal tissues of HCC patients (*P* = 0.021, Table 1). And RAC1 overexpression was positively correlated with the TNM stage of HCC patients (*P* = 0.011, Table 2).

We further analyzed the inter-correlations of ANXA5 with RAC1 and CRKI/II separately, and the correlation of RAC1 and CRKI/II in HCC progression. As the results shown in Table 3, positive correlation was observed for ANXA5 and CRKI/II (*P* = 0.009, *R* = 0.512, Table 3), ANXA5 and RAC1 (*P* = 0.0003, *R* = 0.761, Table 3), and CRKI/II and RAC1 (*P* = 0.001, *R* = 0.461, Table 3). These results implicated ANXA5 might regulate HCC progression and metastasis via CRKI/II and RAC1.

**Table 3:** The positive correlations of the expression levels of ANXA5 with CRKI/II and RAC1, and RAC1 with CRKI/II

Case		ANXA5					*P*
		**−**	**+**	**++**	**+++**	**Total**	
CRKI/II	**−**	2	1	0	0	3	0.009 *R* = 0.512
	+	3	3	1	0	7	
	++	2	7	16	8	33	
	+++	0	1	1	1	3	
	Total	7	12	18	9	46	

Case		ANXA5					*P*
		**−**	**+**	**++**	**+++**	Total	
RAC1	**−**	1	0	0	0	1	0.0003 *R* = 0.761
	+	0	1	0	0	1	
	++	6	11	8	0	25	
	+++	0	0	10	9	19	
	Total	7	12	18	9	46	

Case		**CRKI/II**					*P*
		**−**	**+**	**++**	**+++**	Total	
RAC1	**−**	1	0	0	0	1	0.001 *R* = 0.465
	+	0	1	0	0	1	
	++	1	6	18	0	25	
	+++	1	0	15	3	19	
	Total	3	7	33	3	46	

### Construction of ANXA5 stable knockdown murine hepatocarcinoma Hca-P cell line

**Fig. 2:**
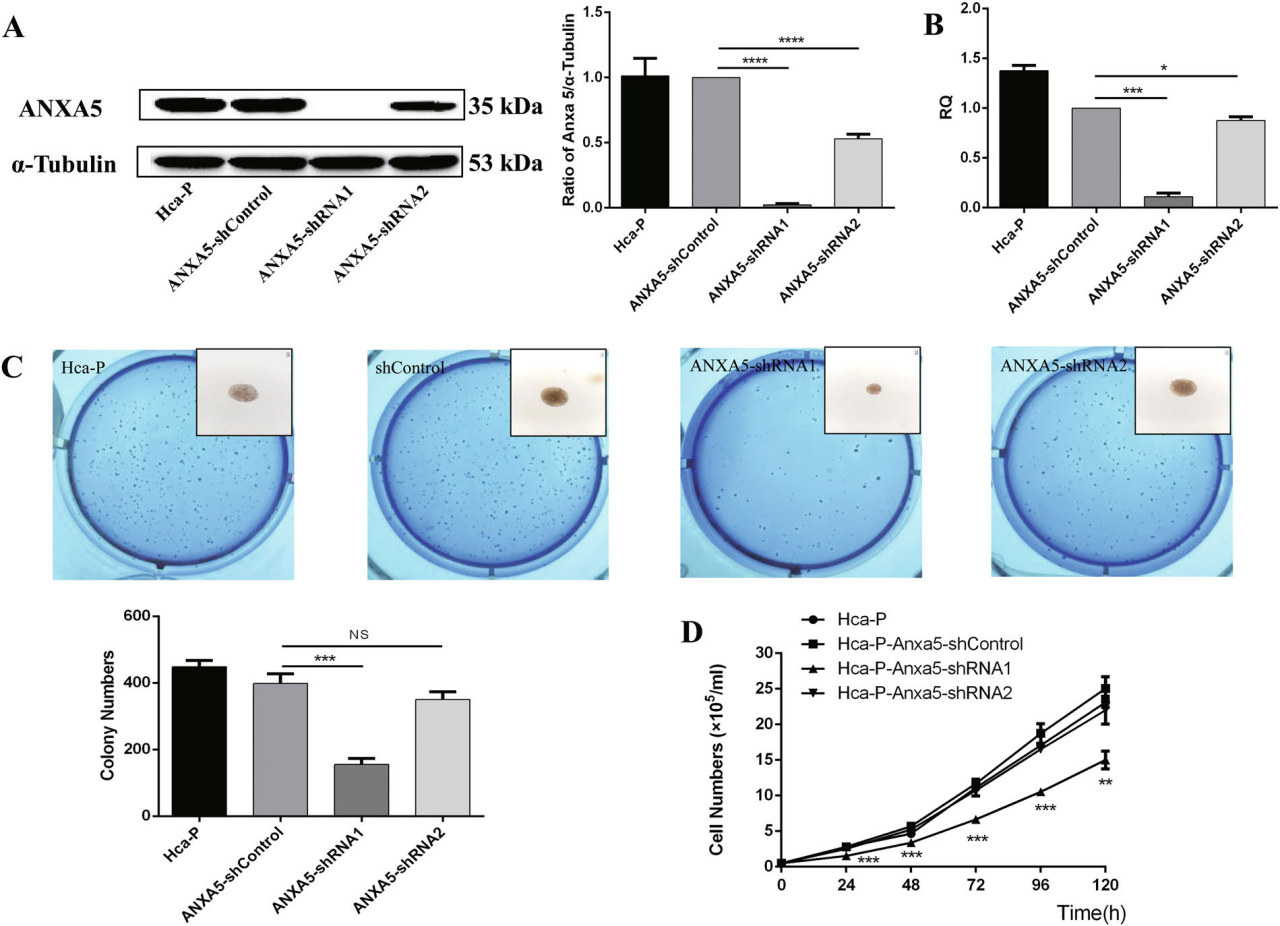
The influence of stable knockdown of ANXA5 on the cell growth of Hca-P cells. **a** Western blotting assay indicated that ANXA5 was down-regulated by ~99% (*P* < 0.001) and ~53% (*P* < 0.001) in Hca-P-Anxa5-shRNA1 and -shRNA2 than the control group cells. Levels are normalized to α-Tubulin. Values mean ± S.D. of three independent experiments. **b** Compared with Hca-P-Anxa5-shControl, qRT-PCR assay showed *Anxa5* levels decreased by ~91% (*P* = 0.0008) and ~17% *(P* = 0.0455) in Hca-P-Anxa5-shRNA1 and -shRNA2 cell lines. **c** The inhibition of ANXA5 knockdown on the colony forming capacity of Hca-P depending on its knockdown extent. Compared with Hca-P-Anxa5-shControl cells, the colony forming activity of Hca-P-Anxa5-shRNA1 decreased by 65.5% (*P* = 0.0003), while, no stastical difference was between Hca-P-Anxa5-shControl and -shRNA2. **d** MTT assay for the effect of ANXA5 knockdown on the proliferation of Hca-P cells. The proliferation decreases of Hca-P-Anxa5-shRNA1 compared with Hca-P-Anxa5-shControl were of stastical significations (*P* < 0.01). No proliferation differences were observed among Hca-P, Hca-P-Anxa5-shControl and -shRNA2. Values mean ± S.D. of three independent experiments

We obtained and selected Hca-P-ANXA5-shRNA1, Hca-P-ANXA5-shRNA2, and Hca-P- ANXA5-shControl monoclonal cell lines, by G418 screening combined with limited dilution method, to study ANXA5 dynamic change on HCC progression and metastasis. No expression changes were determined either at protein (Fig. 2a) and mRNA (Fig. 2b) levels for Anxa5 between Hca-P and Hca-P-ANXA5- shControl cells. Compared with Hca-P-ANXA5-shControl, the ANXA5 protein and *Anxa5* mRNA levels decreased by ~99% (*P* < 0.001, Fig. 2a) and ~91% (*P* = 0.0008, Fig. 2b) in Hca-P-ANXA5-shRNA1 and by ~53% (*P* < 0.001, Fig. 2a) and ~17% (*P* = 0.0455, Fig. 2b) in Hca-P-ANXA5-shRNA2 cells. The monoclonal Hca-P cells with stable Anxa5 knockdowns make it feasible for studying the role of ANXA5 in hepatocarcinoma tumorigenesis and lymphatic metastasis.

### ANXA5 correlates with Hca-P cell growth

**Fig. 3:**
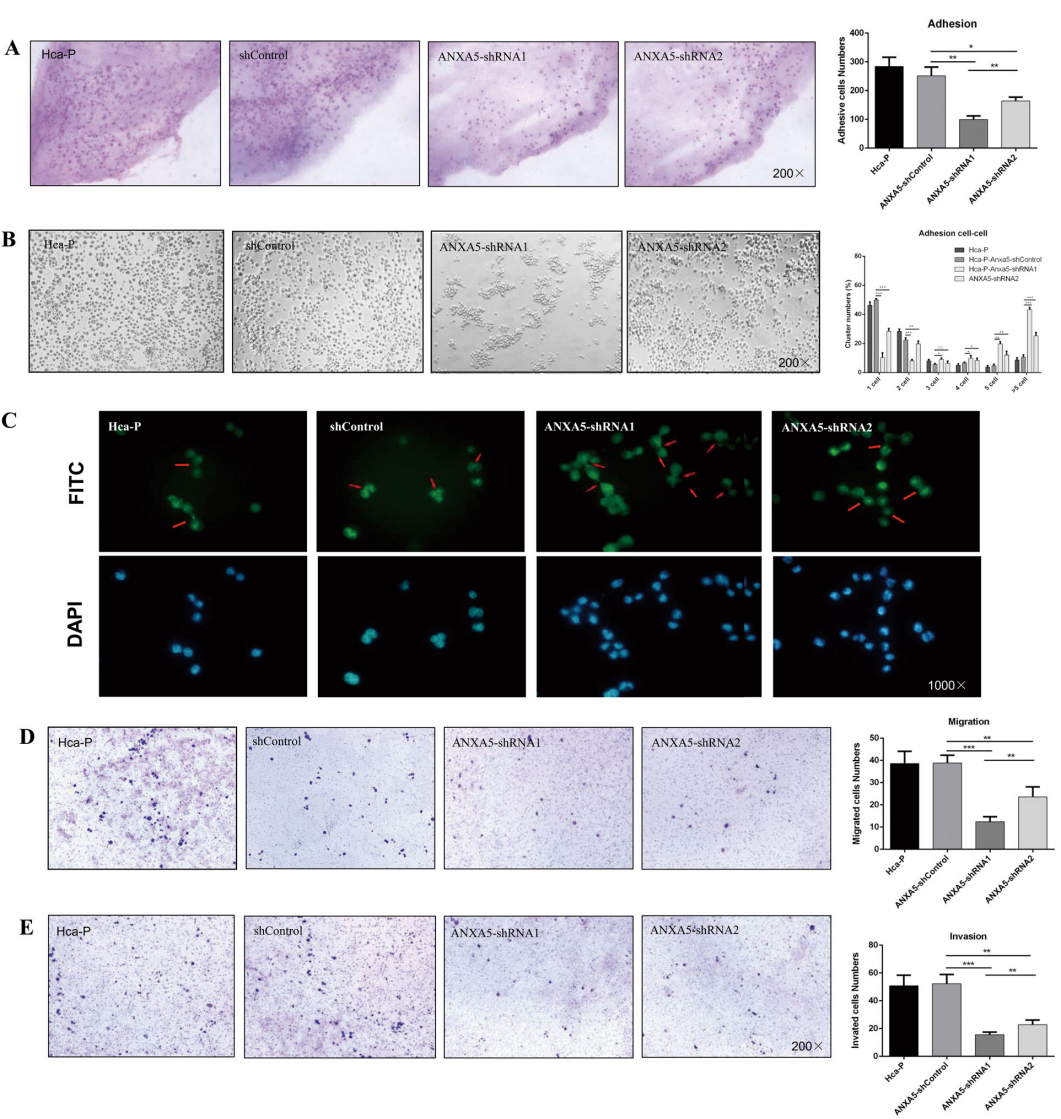
ANXA5 suppression on the LN, cell–cell adhesion, migration, and invasion abilities of Hca-P. **a** The in situ adhesion ability of Hca-P to peripheral LN was decreased following ANXA5 inhibitions (*P* < 0.01). **b** ANXA5 suppression alters growth status of Hca-P. Hca-P-ANXA5-shRNA1 and shRNA2 grows close together easily, whereas Hca-P-ANXA5-shControl cells growing dispersively. Hca-P-ANXA5- shControl cells grow dominantly and evenly with one and two cells* per* aggregate. Following ANXA5 suppression, Hca-P-ANXA5-shRNA1 and -shRNA2 cells grow dominantly with 5 and > 5 cells *per* aggregate. **c** Phalloidin cytoskeleton staining assay of the morphology and structural changes of Hca-P cells following ANXA5 suppression. Its knockdown enhances intercellular adhesion ability of Hca-P. Following ANXA5 suppression, **d** the in vitro migration abilities and **e** invasion capacities of Hca-P-ANXA5-shRNA1 and Hca-P-ANXA5-shRNA2 cells decreased by ~69% (*P* = 0.0003) and 39% (*P* = 0.0026), and decreased by ~71% (*P* = 0.0002) and ~56% (*P* = 0.0018) than Hca-P-ANXA5-shControl cells, respectively. Data are expressed as mean ± S.D. of three experiments

The suppression of ANXA5 on Hca-P growth was related with its downregulation extent. For colony forming capacity, a colony was defined as over 30 cells originally derived from a single cell. During a 10-day-colony-growth incubation, the colonies for Hca-P-ANXA5-shRNA1 and Hca-P-ANXA5-shRNA2 were less than the control, Fig. 2c. Compared with Hca-P-ANXA5-control, Hca-P-ANXA5-shRNA1 showed a decreased colony forming ability by 65.5% (*P* = 0.0003, Fig. 2c). Interestingly, the partial downregulation of ANXA5 expression (~53%) didn’t affect the colony forming ability of Hca-P cells, in fact no apparent difference was measured between Hca-P-ANXA5-shControl and Hca-P-ANXA5-shRNA2 (*P* > 0.05, Fig. 2c). Moreover, the colony size of Hca-P-ANXA5-shRNA1 was smaller than Hca-P, Hca-P-ANXA5- shControl and Hca-P-ANXA5- shRNA2 cells (Fig. 3c).

Trypan blue staining assay on the influence of ANXA5 downregulation on in vitro Hca-P proliferation was consistent with its effect on Hca-P colony forming capacity. As shown in Fig. 2d, no clear differences were measured for the proliferations among Hca-P-ANXA5-shRNA2, Hca-P-ANXA5-shControl and Hca-P. While, compared with Hca-P-ANXA5-shControl, the proliferation of Hca-P-ANXA5- shRNA1 was obvious decreased by ANXA5 knockdown. The suppression of ANXA5 knockdown on Hca-P growth relying on its downregulation extent.

### ANXA5 knockdown attenuates Hca-P adhesion to LN and promotes cell–cell adhesion

ANXA5 deregulation affects the in situ adhesion ability of Hca-P to peripheral lymphatic endothelium. The adhesion abilities (numbers) of Hca-P-ANXA5-shRNA1 and Hca-P-ANXA5-shRNA2 to LN decreased by ~61% ( *P*= 0.0014) and 35% (*P* = 0.0109) than Hca-P-ANXA5-shControl cells (Fig. 3a). A higher degree knockdown of ANXA5 led to a more reduced in situ adhesion of Hca-P to LN.

We then evaluated the effect of ANXA5 knockdown on the intercellular adhesion property of Hca-P cells by hanging drop aggregation assay. The transfection of Hca-P with mock vector did not alter the cell cluster distributions. Hca-P-ANXA5-shControl group cells was dominated with 1 and 2 cells *per* aggregate ( *P* <  0.001, Fig. 3b). Controversially, following ANXA5 knockdown in Hca-P cells, the aggregates of Hca-P-ANXA5-shRNA1 and -shRNA2 were dominated with 5 and >5 cells (*P*   < 0.001, Fig. 3b). The percentage of Hca-P-ANXA5-shRNA1 and -shRNA2 with  >5 cells *per* aggregate increased by ~4 and 2.5 times than Hca-P-ANXA5- shControl, respectively ( *P*<  0.001, Fig. 3b). The number of aggregate cell clusters were more with more ANXA5 knockdown extent.

Phalloidin cytoskeleton staining assay was performed to detect the morphology changes and structural evidences for increased intercellular adhesion ability of Hca-P cells following ANXA5 knockdown. More spreading lamellipodia (sheet-like protrusions from a cell) and more junction sites were found formed at the surfaces of Hca-P-ANXA5-shRNA1 and -shRNA2 cells than Hca-P-ANXA5-shControl cells (Fig. 3c). DAPI staining assay clearly showed more cells were included in each aggregate of Hca-P-ANXA5-shRNA1 and -shRNA2 than Hca-P-ANXA5-shControl cells (Fig. 3c). ANXA5 knockdown enhances intercellular adhesion ability of Hca-P cells.

### ANXA5 knockdown reduces the in vitro migration and invasion of Hca-P

ANXA5 knockdown significantly inhibits the in vitro migration and invasion abilities of Hca-P. ANXA5 knockdown suppressed the in vitro migration ability of Hca-P (Fig. 3d). The numbers of migrated Hca-P-ANXA5-shRNA1 and Hca-P-ANXA5-shRNA2 cells were ~31% (*P* = 0.0003) and 61% (*P* = 0.0026) of that of Hca-P-ANXA5-shControl cells. ANXA5 downregulation also suppressed the in vitro invasion capacity of Hca-P (Fig. 3e). More extent knockdown of ANXA5 resulted in more decreased migration of Hca-P cells.

### ANXA5 knockdown suppresses murine tumorigenicity induced by Hca-P-implantation

**Fig. 4:**
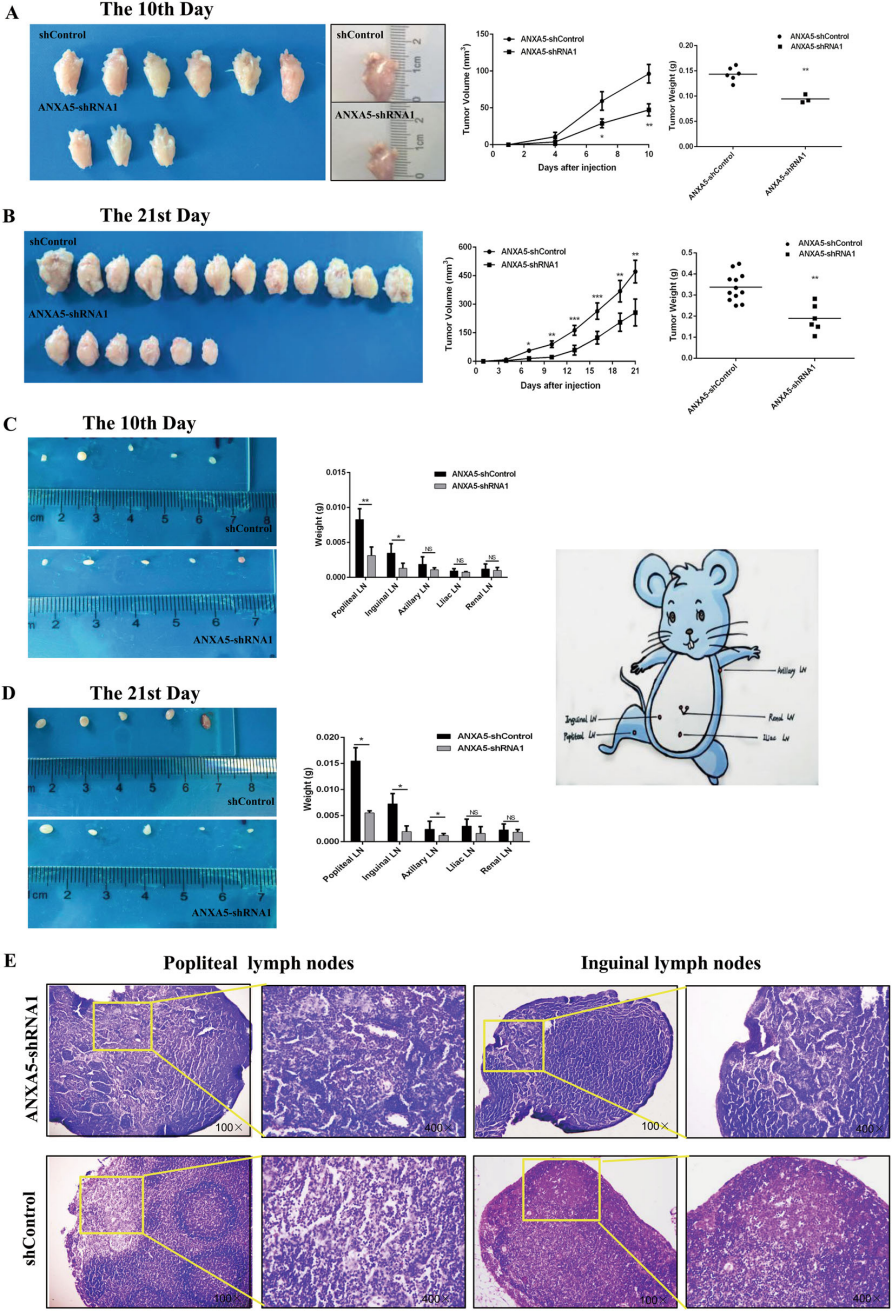
ANXA5 suppression reduces in vivo tumorigenicity and LNM rate of Hca-P. The influence of ANXA5 suppression on in vivo tumor formation volume and mass for mice transplanted with Hca-P-ANXA5-shControl and Hca-P-ANXA5-shRNA1 cells in 10 (**a**) and 21 (**b**) days. Tumor volume (V) was calculated as length × width × depth × π/6. Tumorigenicity of Hca-P-ANXA5-shRNA1 was decreased than Hca-P-ANXA5-shControl with ^**^P < 0.01. ANXA5 suppression decreases LNM malignancy and rate of tumor cell-bearing mice. The mass reductions of popliteal and inguinal LNs from Hca-P-ANXA5-shRNA1-tansplanted mice in **c** 10 and **d** 21 d following transplantation are of stastical significance than control group mice (*P* < 0.05). Data are expressed as mean ± S.D. **e** Popliteal and inguinal LNs with H.E staining. There were few morphological changes of the Hca-P-ANXA5-shRNA1-transplanted mice, including the chromatin condensed in border and the pathological karyokinesis reduced obviously than Hca-P-ANXA5-shControl-transplanted mice. The metastatic LNs from Hca-P-ANXA5-shControl-transplanted mice became more vague and abnormal on 21^st^ d than LNs from Hca-P-ANXA5-shRNA1-transplanted mice

ANXA5 knockdown reduced the in vivo tumorigenicity of mice induced by Hca-P introduction. As the results shown in Fig. 4a, b, all of 18 mice inoculated with Hca-P-ANXA5-shControl cells, while only 3 (Figs. 4a) and 6 (Fig. 4b) mice with Hca-P-ANXA5-shRNA1 cells, formed solid tumors on the 10^th^ and 21^st^ days, respectively. The tumorigenicity ratio of Hca-P-ANXA5-shRNA1-transplanted mice was only 50% of that from Hca-P-ANXA5-shControl-transplanted group mice (Fig. 4a, b). Moreover, the tumor sizes and masses of Hca-P-ANXA5-shRNA1-transplanted mice were much smaller and lighter than Hca-P-ANXA5-shControl-transplanted mice. ANXA5 downregulation suppresses the in vivo murine tumorigenesis of Hca-P cells (Fig. 4a, b).

### ANXA5 downregulation reduces the in vivo LNM rate of Hca-P transplanted mice

To study the role of ANXA5 in tumorigenesis and lymphatic metastasis, the lymph nodes (LNs) from inguinal, popliteal, axillary, iliac arterial and pararenal of the tumor-bearing mice were collected in 10 (soon after tumor formation) and 21days. As shown in Fig. 4c, d, the average masses of popliteal, inguinal, axillary LNs of Hca-P-ANXA5-shRNA1 group were lighter than Hca-P-ANXA5-shControl group. Histological examination (H.E. Staining) shows (Fig. 4e) the number of LNs invaded with Hca-P-ANXA5-shRNA1 cells was less than that of LNs invaded with Hca-P-ANXA5-shControl cells. The structure of the metastatic LNs from Hca-P-ANXA5-shControl-tansplanted mice became vague on 21^st^ day, nevertheless, the structure of LNs from Hca-P-ANXA5-shRNA1-transplanted mice were still distinct with clear and deep nucleus staining (Fig. 4e). ANXA5 knockdown reduces LNM potential of Hca-P cells in the development and progression of tumorigenicity.

### ANXA5 downregulation reduces tumorigenesis of Hca-P-transplanted mice *via* CD34 and VEGF-3

**Fig. 5:**
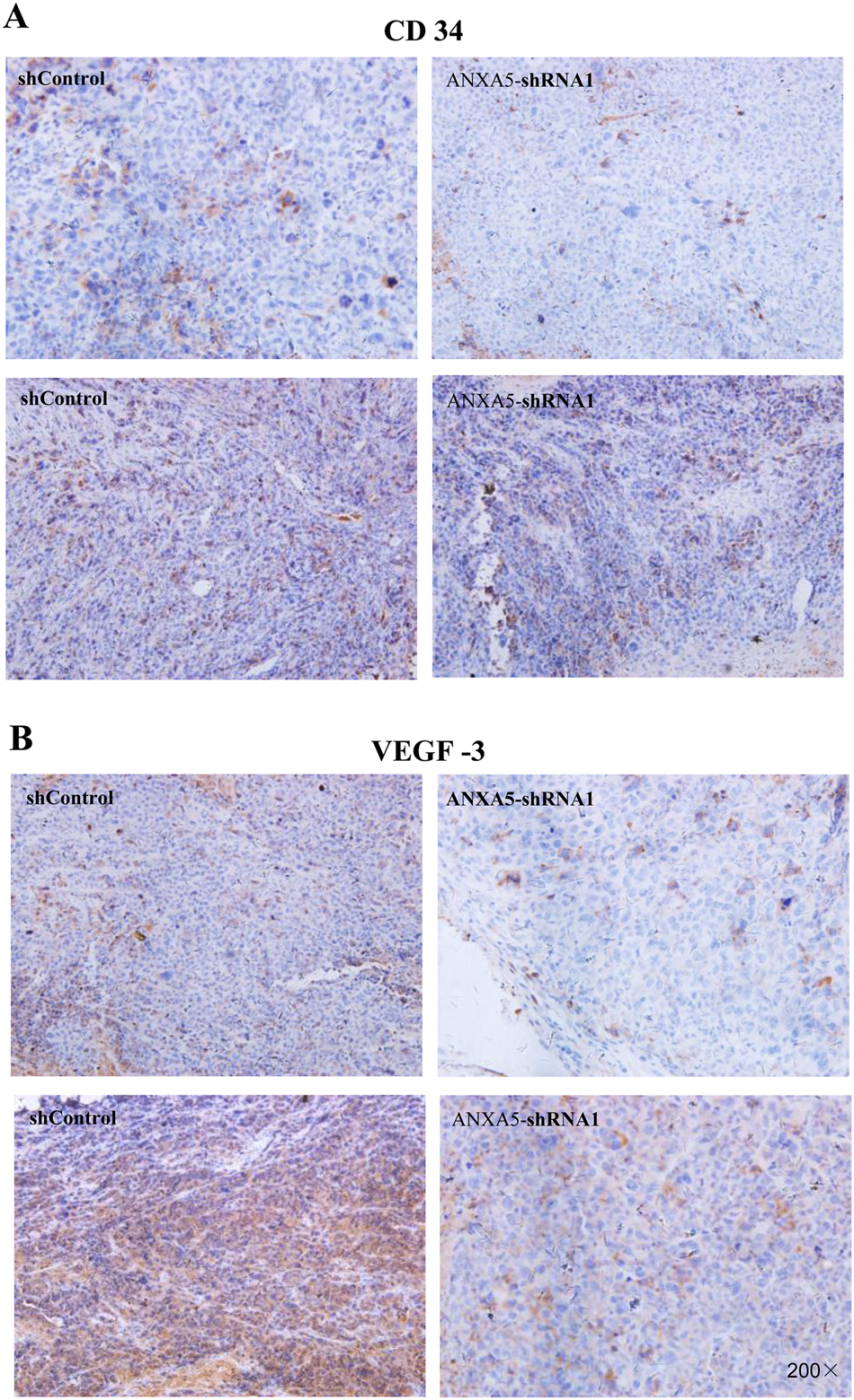
ANXA5 influences tumor formation and malignancy of Hca-P-bearing mice *via* CD34 and VEGF-3 in tumor body. CD34 level comparisons in primary tumors induced by Hca-P-ANXA5-shRNA1 and Hca-P-ANXA5-shControl transplantation in 10 and 21 d (**a**). CD34 expression decreased in tumors from Hca-P-ANXA5-shRNA1 bearing mice, which was more significant in 21 days following transplantation (*P*= 0.002, Table 4). VEGF3 changes in tumors from Hca-P-ANXA5-shRNA1- transplanted mice than Hca-P-ANXA5-shControl group in 10 and 21 d (**b**). Its level decreased in tumors from Hca-P-ANXA5-shRNA1 mice, which was more clear in 21 d following transplantation (*P* = 0.002, Table 4)

ANXA5 knockdown positively correlated with reduced levels of CD34 and VEGF-3, two angiogenesis and lymphangiogenesis indicators, in suppressing Hca-P-transplantation induced murine tumorigenesis. CD34 and VEGF-3 levels in primary tumors collected from Hca-P-ANXA5-shRNA1- and Hca-P-ANXA5-shControl-transplanted mice on 10^th^ (soon after tumor formation) and 21^st^ d were compared by IHC assay (Fig. 5).

CD34 was locally expressed in cell membrane and cytosol. Although without statistical significance (Fig. 5a, Table 4, *P*= 0.72), CD34 expression level in primary tumor tissues from Hca-P-ANXA5-shRNA1 group mice on the 10^th^ day following cell implantation showed a reduction tendency than Hca-P-ANXA5-shControl group mice. On 21^st^ day, CD34 expression was depressed in the tissues from the Hca-P-ANXA5-shRNA1 group mice significantly (Fig. 5a, Table 4, *P* = 0.002).

**Table 4:** CD34 /VEGF3/AXNA5/CRKI/II/RAC1 expression decreased in tumors from Hca-P-ANXA5-shRNA1 bearing mice

Molecule	Group	The 10^th^ Day				*P*	The 21^st^ Day				*P*
		−	+	++	+++		−	+	++	+++	
CD34	Hca-P-Anxa5-shControl	2	2	1	1	0.072	0	1	5	6	0.002
	Hca-P-Anxa5-shRNA1	5	1	0	0		8	2	2	0	
VEGF3	Hca-P-Anxa5-shControl	1	3	2	0	0.21	1	0	4	7	0.002
	Hca-P-Anxa5-shRNA1	4	1	1	0		7	3	2	0	
ANXA5	Hca-P-Anxa5-shControl	0	1	2	3	0.004	0	2	5	5	0.001
	Hca-P-Anxa5-shRNA1	4	2	0	0		9	3	0	0	
CRKI/II	Hca-P-Anxa5-shControl	2	0	1	3	0.309	1	1	4	6	0.041
	Hca-P-Anxa5-shRNA1	3	1	1	1		5	3	1	3	
RAC1	Hca-P-Anxa5-shControl	0	2	2	2	0.238	0	1	5	6	0.013
	Hca-P-Anxa5-shRNA1	1	3	1	1		1	7	2	2	

VEGF-3 expression was located in cytosol (Fig. 5b, Table 4). Similar result was obtained on the influence of ANXA5 knockdown on VEGF-3 expression in primary tumor induced by Hca-P cell transplantation in mouse.

These findings indicate ANXA5 knockdown suppresses in vivo HCC progression and metastasis via downregulating the expressions levels of CD34 and VEGF-3 in tumorigenesis.

### ANXA5 knockdown potentially downregulated CRKI/II and RAC1 in suppressing tumorigenicity of Hca-P-transplanted mice

**Fig. 6:**
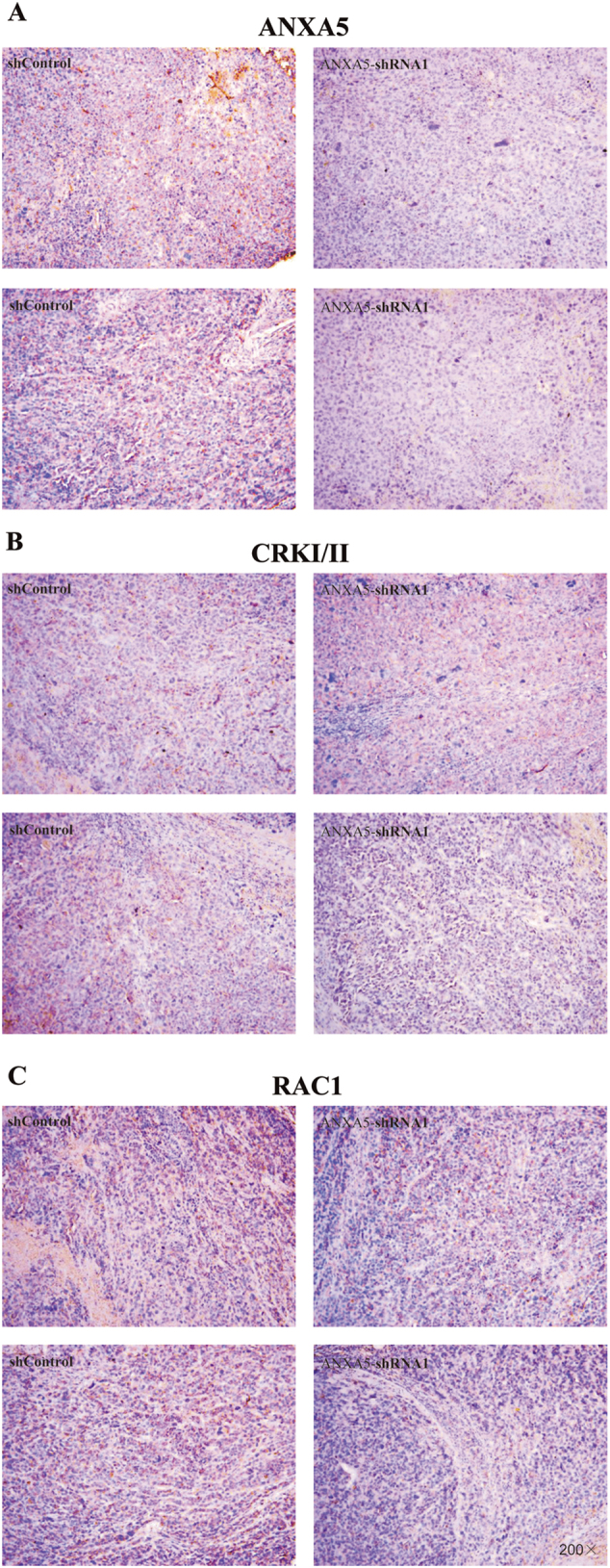
Deregulating ANXA5 reduced the In vivo metastasis of Hca-P transplanted mice through CRKI/II and RAC1. IHC results shown ANXA5 was downregulation in tumor body both in 10 and 21 d (*P*= 0.001, A, Table 4). CRKI/II expression was suppressed in Hca-P-ANXA5-shRNA1 group in 21 d with significantly statistics (*P* = 0.041, B, Table 4). RAC1 expression had a decreased trend in 10d, and clear decrease in 21 d following transplantation (*P* = 0.013, C, Table 4)

ANXA5 knockdown in Hca-P led to reduced expressions of CRKI/II and RAC1 in its induced primary tumors of mice. ANXA5 was distributed in cell membrane and cytosol. ANXA5 expression levels in tumorous tissues on both 10^th^ and 21^st^ d were decreased (Fig. 6a, Table 4, *P* = 0.001).

Although without statistical significance, CRKI/II and RAC1 showed decreased expression tendencies in tumor tissues from Hca-P-ANXA5-shRNA1 than Hca-P-ANXA5-Control group on 10^th^ d (Fig. 6b, c, Table 4, *P* = 0.309). On 21^st^ d following tumor cell implantation, the decreases of CRKI/II and RAC1 expressions in primary tumors of Hca-P-ANXA5-shRNA1 group mice were of statistical significances.

Conclusively, ANXA5 downregulation inhibited hepatocarcinoma cell induced primary tumor growth of mice *via* downregulating CRKI/II and RAC1.

### ANXA5 mediates Hca-P behaviors *via*CRK/DOCK180/RAC1 and pMEK-ERK pathways

Tumor migration and invasion are strictly regulated by actin cytoskeleton modulated by Rho family of small GTPases, including RAC1, Cdc42, and RhoA in space timely. DOCK180 is a guanine-nucleotide exchange factor for mediating RAC1 and Rap1. CRKI and CRKII, two alternative splicing products of signaling adaptor CRK family, delivery signals to GTPases through its downstream effector DOCK180. Based on previous results on the positive correlations of ANXA5 with RAC1 and CRKI/II in HCC progression and metastasis, we proposed and investigated the molecular action mechanism and association of ANXA5 with CRKI/II, RAC1 and DOCK80 using Hca-P cells.

**Fig. 7:**
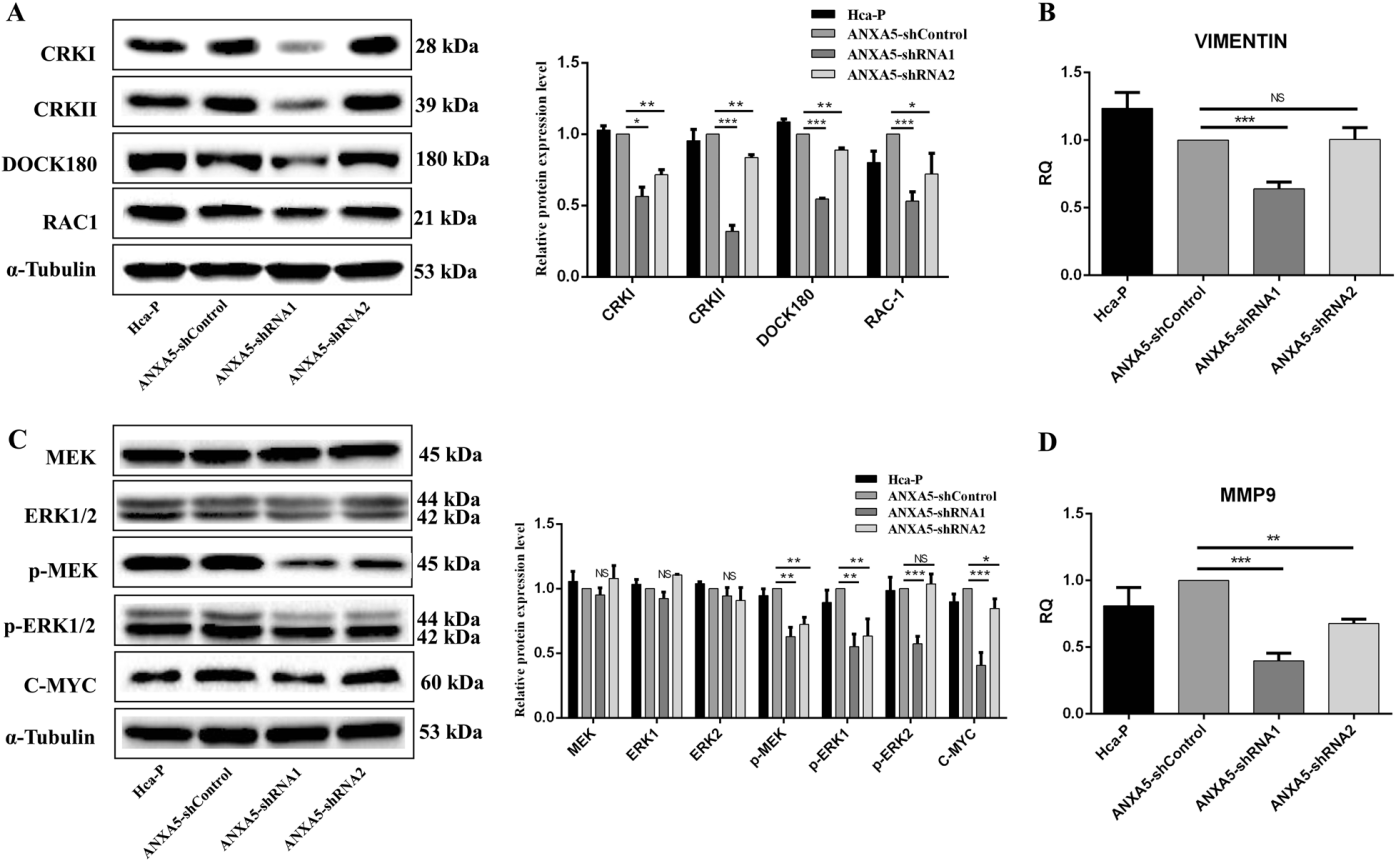
ANXA5 mediates CRK/DOCK180/RAC1 and ERK activities in Hca-P. **a** ANXA5 suppression reduced CRKI, CRKII, DOCK180 and RAC1 in Hca-P. No change was between Hca-P and Hca-P-ANXA5-shControl. Compared with Hca-P-ANXA5-shControl, the different expressions of CRKI, CRKII, DOCK180 and RAC1 in Hca-P-Anxa5-shRNA1 and -shRNA2 were significant (*P* < 0.05). Decreases in Hca-P-Anxa5-shRNA1 were more clear than Hca-P-Anxa5-shRNA2 cells. **b** The influence of ANXA5 downregulation on vimentin. **c** The influence of ANXA5 stable knockdown on critical molecules in ERK pathway. ANXA5 suppression could not alter the levels of MEK, ERK1, and ERK2. Except for p-ERK2 in Hca-P-Anxa5-shRNA2, the decreases of p-MEK (*P* < 0.01), p-ERK1 (*P* < 0.01), p-ERK2 (*P* < 0.001) and C-MYC (*P*< 0.05) in Hca-P-Anxa5-shRNA1 and -shRNA2 cells were significant than those of Hca-P-Anxa5-shControl, and in Hca-P-Anxa5-shRNA1 were more clear than Hca-P-Anxa5-shRNA2 cells. **d** ANXA5 knockdown reduces the expression of MMP-9 in Hca-P-Anxa5-shRNA1 (*P* < 0.001) and -shRNA2 (*P* < 0.01) cells. Levels are normalized to α-Tubulin. Values mean ± S.D. of three independent experiments

ANXA5 inhibition suppresses the malignant behaviors through decreasing the activity of integrin pathway. WB assay indicated the expression levels of critical molecules in the pathway including CRKI, CCRKII, DOCK180 and RAC1 were decreased by complete ANXA5 knockdown. (Fig. 7a, *P* < 0.05). The expression level of VIMENTIN, an EMT marker regulates VAV2-mediated Rac1 activation, reduced by 36% (*P*= 0.0002, Fig. 7b) in Hca-P-ANXA5-shRNA1 than in the control cells. While, comparable mRNA expression level was determined in Hca-P-ANXA5-shRNA2 and -shControl cells (*P* = 0.9096, Fig. 7b). More ANXA5 inhibition lead to more decreased expressions of CRKI, CRKII, DOCK180, Rac1 and VIEMTIN in Hca-P cells.

**Fig. 8:**
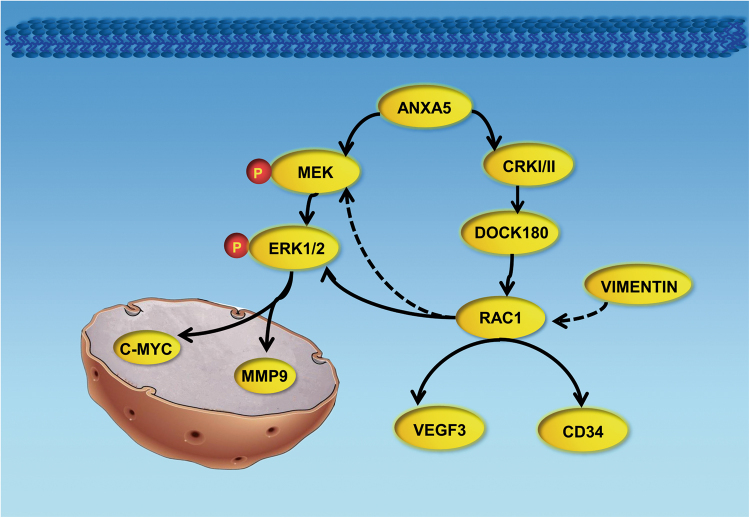
ANXA5 regulates Hca-P cell metastasis trough RAC1/ERK1/2 pathway. ANXA5 knockdown reduced the expression level of VIMENTIN and integrin pathway molecules CRKI, CRKII, DOCK180, and RAC1, as well as ERK pathway molecules p-MEK, p-ERK1 and p-ERK2. The inactive effect on these molecules result in suppressing signal transductions from cell surface into nuclear to invoke MMP9 and C-MYC. CD34 and VEGF-3, two angiogenesis and lymphangiogenesis indicators, was both inhibited by ANXA5 knockdown. All of these changes lead to decreased in vitro proliferation, migration and invasion, and in vivo tumorigenicity and metastasis of hepatocarcinoma cells

We analyzed the influences of ANXA5 inhibition on the MEK/ERK pathway. Following ANXA5 knockdown in Hca-P cells, WB indicated that the expression levels of p-MEK (Ser217/221) and p-ERK1 were both decreased in Hca-P-ANXA5-shRNA1 and Hca-P-ANXA5-shRNA2 cells ( *P*< 0.05), p-ERK2 (Thr202/ Tyr204) was apparently decreased in Hca-P-ANXA5-shRNA1 (*P* < 0.001), while was unchanged in Hca-P-ANXA5-shRNA2 (*P* > 0.05), Fig. 7c. No changes were detectable for the total MEK and ERK levels (Fig. 7c). We then found that the level changes of C-MYC and MMP9, two molecules regulated directly by ERK1/2, reduced by 69 and 60% (*p* < 0.01, Fig. 6c, d) in Hca-P-ANXA5-shRNA1 cells, and by 15% and 32% in Hca-P-ANXA5-shRNA2 (*p* < 0.05, Fig. 7c, d) than control group cells, respectively.

Taken together, ANXA5 inhibition potentially suppresses the critical molecules CRKI, CRKII, DOCK180 and RAC1 in integrin pathway, p-MEK, p-ERK1, and p-ERK2 in ERK pathway, as well as VIMENTIN in EMT for inhibiting signal transduction into nuclear membrane to invoke MMP9 and C-MYC, as summarized and schemated in Fig. 8,  which results in decreased in vitro proliferation, migration and invasion, and in vivo tumorigenicity velocity, malignancy and metastasis of hepatocarcinoma cells.

## Discussion

As a member of annexin A family, Anxa5 plays important roles in vesicle trafficking and cell behaviors^25–27^, in the development, progression and diagnosis of some cancers^6–10^. At present, the research of Anxa5 in cancer lymphatic metastasis is limited. Previous work from our group demonstrated ANXA5 up-regulation increased the in vitro and in vivo malignancy behavior^16^. In current work, we investigated the associations of ANXA5 overexpression with the clinical progression and lymphatic metastasis in hepatocarcinoma patients’ tissue microarray, and the action mechanism in vivo and in vitro.

We found ANXA5 overexpression contributed to the progression and metastasis of HCC patients. It was correlated with TNM stage advance. Our work showed the positive correlations of ANXA5 level with CRKI/II and RAC1, critical molecules in integrin pathway, in tumorous tissues from HCC patients. As two alternative splicing products of v-CRK^28^, CRKI and CRKII have been linked to a variety of human cancers^28–30^. Through DOCK180, CRKI/II was suggested to deliver signals to small GTPases, RAC1, Cdc42 and RhoA, for modulating actin cytoskeleton organization in tumor malignancy^31^. The overexpression of CRKI/II and RAC1 commonly lead to relative higher tumor cell migration and invasions^32–34^. Compared with the adjacent normal tissues, IHC assay showed CRKI/II and RAC1 were apparently overexpressed in HCC tumorous tissues and also positively correlated with patients’ TNM stage. Mutual inter-positive correlations were apparently established for the levels of ANXA5, CRKI/II and RAC1 in tumorous tissues from patients. Interestingly, all of the three protein showed highly expression level in three patients diagnosed with LNM. The above results implicate ANXA5 upregulation that is positively correlated with CRKI/II and RAC1 potentially promotes the clinical progression and metastasis of HCC.

We then investigated its potential action mechanism in HCC carcinogenicity and LNM using Hca-P cell line model. We constructed Hca-P cell lines with ANXA5 stable knockdowns to investigate consequent effects on the in vitro and in vivo malignant behaviors of Hca-P with underlying action mechanism. Compared with Hca-P-ANXA5-shControl, the monoclonal Hca-P-ANXA5-shRNA1 showed more decreased degree of ANXA5 expression than Hca-P-ANXA5-shRNA2 cell lines.

ANXA5 suppression inhibited the in vitro growth, migration and invasion, in situ cell adhesion to LN as well as promoted the in vitro cell cohesion and aggregate capacities of Hca-P related to its knockdown extent. The cohesive and aggregative growth of Hca-P promoted by ANXA5 downregulation was further supported by phalloidin staining assay. The above results suggest ANXA5 might mediate Hca-P malignant properties *via* affecting its adhesion to LNs, and intercellular cohesion and aggregate capacity. Consistently, a greater knockdown extent of ANXA5 led to more clearly significant suppressions on in vitro malignant property and metastasis ability of Hca-P cells.

Then, consistent with the in vitro evidences, we found ANXA5 suppression led to apparently decreased the in vivo tumorigenicity and LNM potential of Hca-P- transplanted mice. The attenuation of angiogenesis and lymphangiogenesis attenuate tumor development and metastasis^35,36^. Current work showed ANXA5 mediated Hca-P-transplantation induced murine tumorigenesis and metastasis *via* CD34 and VEGF-3, two well-known angiogenesis and lymphangiogenesis indicators^37,38^. As ANXA5, CRKI/II and RAC1 expressions were upregulated and positively correlated in tumorous tissues from HCC patients, we then measured ANXA5, CRKI/II and RAC1 expression levels in primary tumors from transplanted mice. The results showed ANXA5, CRKI/II, and RAC1 were reduced in Hca-P-shANXA5-transplanted mice.

CRKI/II usually binds to DOCK180 to activate RAC1^39^, resulting in activated MEK-ERK signaling transduction through PAK to mediates cell migration^40–42^. Hence, we proposed ANXA5 suppression might inhibit Hca-P malignant properties *via* CRK/DOCK180/RAC1 and MEK-ERK pathways. We found CRKI, CCRKII, DOCK180, RAC1, p-MEK (Ser217/221) and p-ERK1 levels reduced in Hca-P -ANXA5-shRNA1 and Hca-P-ANXA5-shRNA2 cells than Hca-P-ANXA5-shControl cells. Interestingly, p-ERK2 (Thr202/Tyr204) level was significantly reduced in Hca-P-ANXA5-shRNA1 cells, while, indistinctly altered in Hca-P-ANXA5-shRNA2 than Hca-P-ANXA5-Control cells. These results helped to understand why Hca-P-ANXA5-shRNA1 showed much more decreased malignant properties than Hca-P-ANXA5-shRNA2 cells. MMP9 and C-MYC are two critical molecules in MEK/ERK cascade-mediated cell migration and proliferation^43,44^. p-MEK phosphorylates both the Ser/Thr and Tyr residues of ERK1/2, transduces signals from cell surface into cytosol and nucleus, results in reduced transcriptional activities of MMP9 and C-MYC^45–48^. Our work showed ANXA5 knockdowns resulted in decreased expressions of C-MYC and MMP9 in Hca-P-ANXA5-shRNA1 and Hca-P-ANXA5-shRNA2 cells. Greater extent suppressions of these molecules were detected in Hca-P-ANXA5-shRNA1 than Hca-P-ANXA5-shRNA2, which consequently contributed in the greater extent suppressions of malignant phenotypes and behaviors of hepatocarcinoma cells, which also implicated the linkage of ANXA5 overexpression positively correlated with RAC1 and CRKI/II in promoting the clinical progression and metastasis of HCC. More scientific and clinical significances for investigating the role and action mechanism of ANXA5 in the progression and metastasis of human cancers and cancer cell lines should be addressed in the further. The new interesting oncotargets such as ANXA5 partners to be identified by high-throughput techniques including shotgun proteomics, comparative genomics, RNA-omics should provide complementary oncotargets to ANXA5 and supply new clues to tumorigenesis and the underlying mechanism.

In conclusion, as summarized and schemed in Fig. 8, ANXA5 overexpression positively correlated with CRKI/II and RAC1 potentially promoted the clinical development and metastasis of HCC as well as tumorigenicity and lymph node metastasis in vivo. ANXA5 downregulation decreased the expressions of CRKI/II, DOCK180, RAC1 in integrin pathway, p-MEK, p-ERK, c-Myc, and MMP-9 in MEK-ERK pathway together with VIMINTIN in Hca-P cells for its reduced malignancy, Fig. 8. Anxa5 is a potential target in the diagnosis and treatment of HCC.

## Materials and methods

### Cell culture, experimental animal and HCC tissue array

Hca-P, a murine hepatocarcinoma cell line with ~25% lymph node metastasis (LNM) rate was established and maintained in our research group at Dalian Medical University. 2 × 10^6^ Hca-P cells were implanted into the abdominal cavity of 615-mouse (aged 6 w, 20 ± 2 g; Certificate number: SCXK (Liao) 2008-0002). The Hca-P cells taken from mouse abdominal cavity were purified *via* 2 passages and cultured in RPMI 1640 (Gibco, USA) with 15% FBS at 37 °C with 5% CO_2_. Experimental mice supplied by the SPF Animal Laboratory Center of Dalian Medical University were fed, treated and sacrificed according to approved protocols by Experimental Animal Ethical Committee of Dalian Medical University (Permit Number: L2012012).

HCC tissue microarray was from Super Biotek (Shanghai, China). It was composed of 46 cases of HCC tumorous tissues including 3 HCC tissues with lymphatic metastasis.

### IHC assay

IHC assay was done to determine the expression changes of ANXA5 (Proteintech, China), RAC1 (Proteintech, China) and CRKI/II (GeneTex,USA) in HCC tissue array, and of CD34 and VEGF-3 (SantaCruz, USA) in primary tissues from Hca-P-ANXA5-shRNA1 and Hca-P-ANXA5-shControl-transplanted mice. Tissue sections were treated with biotin-streptavidin HRP detection systems (ZSGB-BIO, China) according to the manufacturer’s protocol. The images were visualized with 3,3′-diamino-benzidine (DAB) kit (ZSGB-BIO, China) under an BX3-CBH microscope (Olympus, Japan).

IHC immunoreaction intensity was rated into four grades, 0 (negative), 1 (weak), 2 (moderate), and 3 (strong) as Score I. Moreover, based on the detected positively staining cells, DAB staining quantity of each sample was graded as 0 (none), 1 (1–10% cells per field), 2 (10–50%), 3 (51–75%) and 4 (>76%) as Score II. The multiplication of Score I by Score II ranged 0 to 12 was used for IHC immunoreactivity degree. The scores of 0–2, 3–5, 6–8, and 9–12 were considered as negative (−), weak (+), moderate (++) and strong (+++). IHC assay was scored by two independent experienced pathologists, separately.

### Establishment of monoclonal Hca-P cell line with stable ANXA5 knockdown

Two *Anxa5* (Genbank: BC003716.1) targeting shRNAs, shRNA1 and shRNA2 were designed using siDirect and Whitehead softwares. The plasmids shRNA1, shRNA2 and a negative control shRNA for ANXA5 were inserted into pGPU6/GFP/ Neo vectors and transfected into Hca-P cells using Lipofectamine^TM^ 2000 (Life Technologies, USA) following our procedures^17^. The monoclonal Hca-P-ANXA5-shRNA1, -shRNA2, and -shControl cell lines were screened against 200 μg/ml G418 using a 96-well plate *via* limited dilution method.

### Quantitative real-time PCR (qRT-PCR) assay

qRT-PCR assay was performed for analyzing the level changes of *Anxa5, MMP9*, and *Vimentin* among different group cells using GAPDH as internal reference. Total RNA was extracted from each group cells using Trizol^TM^ reagent (Life Technologies, USA). cDNA was prepared with Primescript RT Reagent (TaKaRa, Japan). Quantitative real-time PCR (qRT-PCR) was carried out on an ABI Step One Plus Real -Time-PCR System (Applied Biosystems, USA) with FastStart Universal SYBR Green Master (ROX) (Roche, Germany). Primers for targeting molecules were designed as below: *Anxa5*, F: 5′-CATCTTTGGGACACGCAG-3′, R: 5′-GGTCAAT CTCACTCCTC-3′; *MMP9*, F: 5′-CCAGAGGTAACCCACGTCAGC-3′, R: 5′-TTTG GAAACTCACACGCCAGA-3′; *Vimentin*, F: 5′-GACATTGAGATCGCCACCT-3′, R:5′-ATCTCTGGTCTCAACCGTCT-3′; *GAPDH*, F: 5′- GGTGAAGGTCGGTGTGAACG-3′, R: 5′-CTCGCTCCTGGAAGATGGTG-3′.

### SDA–PAGE and Western blotting assay

The total protein extraction and western blot were performed, as previously described^17^. The primary antibodies were ANXA5 (1:3000, Proteintech, China), p-ERK1/2 (Thr202/Tyr204) (1:1000, Cell Signaling Technology, USA), p-MEK (Ser217 /221) (1:1000, Cell Signaling Technology, USA), DOCK180 (1:400, Proteintech, China), RAC1 (1:800, Proteintech, China), C-MYC (1:800, Proteintech, China), α-Tubulin (1:5000, Proteintech, China), CRKI/II (1:1000, GeneTex, USA). Protein bands were visualized by ECL (Adavansta, USA) and imaged by Bio-Rad ChemiDoc^TM^ MP system (Bio-Rad, USA).

### Cell growth assay

For colony forming, the base layer was prepared by mixing 1 mL 1.2% melted soft agar (AMRESCO, USA) with 1 mL 2 × medium (15%FBS in RPMI-1640). 1 mL 0.7% soft agar was mixed with 1 mL 2 × medium (15% FBS in RPMI-1640), mixed well with 1000 cells from each group, and seeded evenly onto the top of the basal layer as the upper layer in a 6-well plate. The plates were then incubated with 5% CO_2_ at 37 °C for 10 d. Cell colonies were fixed in methyl hydrate for 10 min and stained in 0.1% crystal violet, counted using a microscope and compared. For trypan blue staining assay, 1 mL (5 × 10^4^cells/mL) different group cells were added into 24-well plate and incubated at 37 °C with 5% CO_2_. Surviving cells were counted using a cytometer with trypan blue staining every day for five consecutive days.

### In situ cell adhesion potential to lymph node (LN) assay

Frozen fresh LNs from 615 mice were sectioned into 10 µm slices. The surfaces of frozen slices of inguinal and popliteal LNs were covered in 200 μL serum-free RPMI 1640 containing 2 × 10^5^ cells of Hca-P, Hca-P-ANXA5-shRNA1, -shRNA2, and shControl at 37 °C with 5% CO_2_ for 24 h, fixed in 95% ethanol, washed with flowing distilled water for 3 s and stained with hematoxylin eosin (HE). The adherent cells was counted and averaged from five random fields using a microscope (Olympus, Japan).

### Hanging drop aggregation assay for intercellular adhesion

The effect of ANXA5 knockdown on the cell–cell adhesion ability of Hca-P cells were determined by hanging drop aggregation assay^48^. In total 30 μL of medium containing 5000 cells was loaded onto the inner surface of the cover of a 100 mm diameter Petri dish and incubated the hanging drop cells overnight at 37 °C with 5% CO_2_. The number of cells in cell aggregates were carefully counted by two investigators, separately, using an inverted microscope (Olympus, Japan) by randomly selecting 100 attached sites. Triplicate experiments were performed for each assay.

### Phalloidin cytoskeleton staining assay for cellular morphology

In total 2 × 10^5^ cells in 200 μL RPMI-1640 with 15% FBS was loaded onto the center of a glass slide, put into a wet box, incubated in a humidified environment at 37 °C with 5% CO_2_ for 24 h. The cells were then anchored in 4% paraformaldehyde solution for 10 min, and dehydrated with pre-cool acetone (−20 °C) for 5 min. Next incubated with 400 nM FITC-phalloidin (Sigma, USA) for 30 min in darkness at RT. The slides were counterstained in 100 μL 10 μg/ml Hoechst 33258 (Sigma, USA) for cell nucleus. Images were taken by a fluorescence microscope (Olympus, Japan) with ×1000.

### In vitro cell migration and invasion assays

The 24-well transwell units with 8 mm I.D. polyester membrane (Corning, USA) were employed. For migration assay, 600 μL RPMI-1640 with 15% FBS and 6 × 10^4^ cells from each group in 200 μL RPMI-1640 were loaded into the lower and upper chambers respectively, and incubated at 37 °C with 5% CO_2_ for 24 h. The cells migrated to lower surface of the filter were fixed in 4% paraformaldehyde for 20 min, stained in 0.1% crystal violet for 20 min, counted by randomly selecting 5 fields per well with a light microscope at a magnification of ×200.

For invasion assay, the fitter surface of an insert transwell unit was first coated with 50 μL ice-cold ECM gel (1:5 dilution with RPMI 1640, Sigma-Aldrich, USA) by incubating at 37 °C for 8 h. The rest steps were the same as in migration assay.

### In vivo tumorigenicity and LNM investigations

Thirty-six 615 mice (4–6 w, 21 ± 2 g), half male and half female, were used for in vivo tumorigenicity subjects of Hca-P cells. These mice were randomly divided into two groups with 18 mice* per* group, half male and half female. 2 × 10^6^ of Hca-P-ANXA5-shControl and Hca-P-ANXA5-shRNA1 cells in 25 μL medium were transplanted into the footpads of mice. The length, width, depth of primary tumor formed on the footpad was measured using a vernier calliper. Tumor volume (V) was calculated according to the formula: V = length × width × depth × π/6. The primary neoplastic tissues and LNs from the inguinal, popliteal, axillary, iliac arterial, pararenal of the tumor-baring mice were isolated, weighted and photographed on 10^th^ and 21^st^ d following tumor cell implantation. The tissues were fixed in neutral formalin for 48 h and embedded in paraffin for later use.

### Data processing and statistical analysis

The data were presented as mean ± SD of at least three independent experiments. Comparisons between two groups were performed by Student’s *t*-test. For IHC assay, a Mann–Whitney U or Fisher exact test and Spearman rank correlation was employed. SPSS17.0 was used for statistical analysis. Results with *P* < 0.05 were of significance.

## Electronic supplementary material

Supplemental figures
