# Supplementary material for: Annexin A5 regulates hepatocarcinoma malignancy via CRKI/II-DOCK180-RAC1 integrin and MEK-ERK pathways
Source: Cell Death Dis. 2018 May 25;9(6):637. doi: 10.1038/s41419-018-0685-8 (PMC5970249; doi:10.1038/s41419-018-0685-8)
Supplement: Supplementary file 1 — Supplemental figures [file 41419_2018_685_MOESM1_ESM.docx]

SUPPLEMENTARY FIGURES


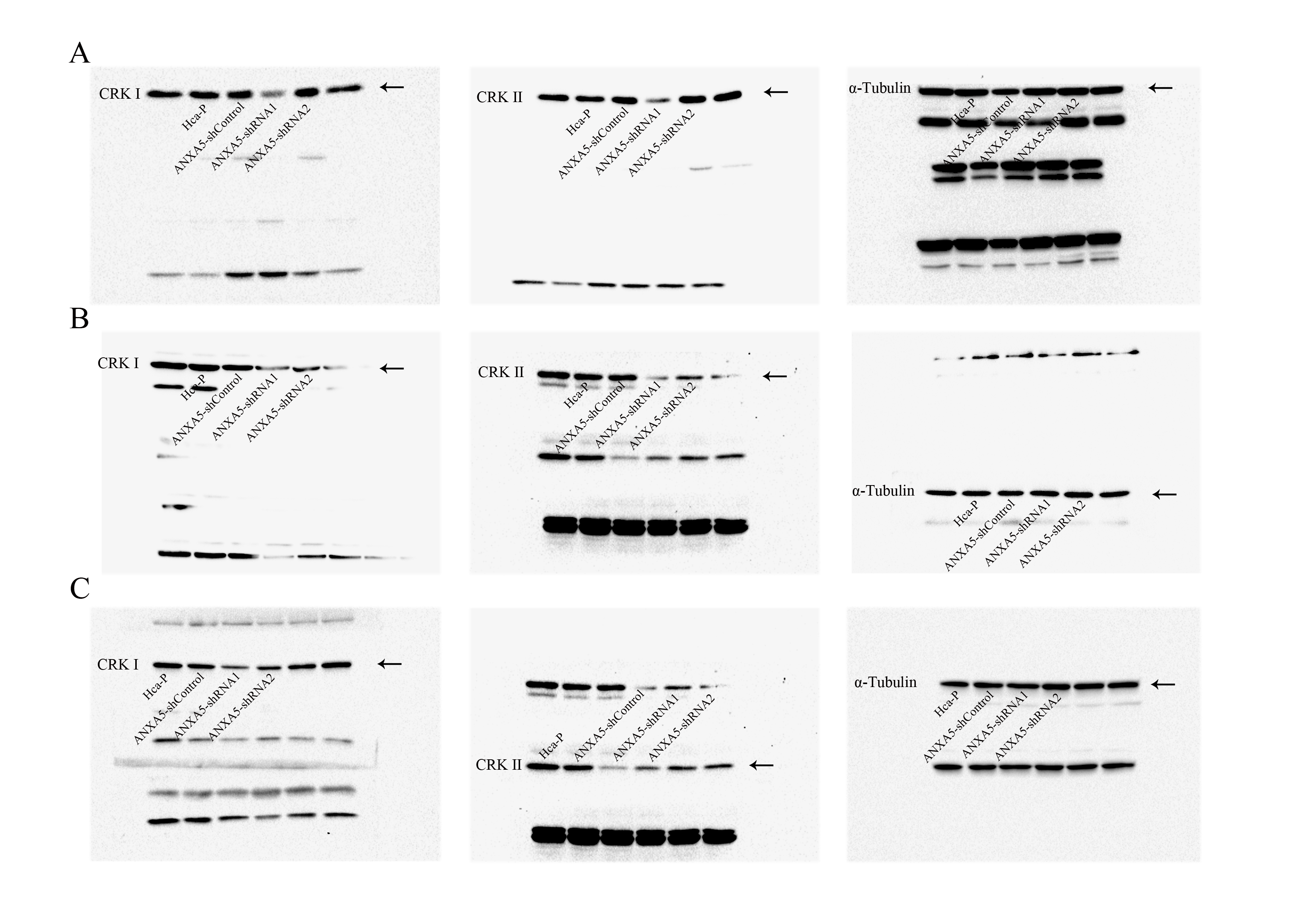


53

39

28

**Figure S1: The protein expression levels of CRKI, CRKII in Hca-P, Hca-P-ANXA5-shControl, Hca-P-ANXA5-shRNA1 and Hca-P-ANXA5-shRNA2 cells** **were detected by Western blotting assay.** ANXA5 knockdown reduced CRKI, CRKII expression in Hca-P. (A) The first, (B) second and (C) third uncropped scans blots of CRKI, CRKII and α-tubulin were from triplicate independent experiments. α-tubulin was used as the internal reference for relative quantification. The relative protein levels were then quantified using the grey intensity difference analysis method.


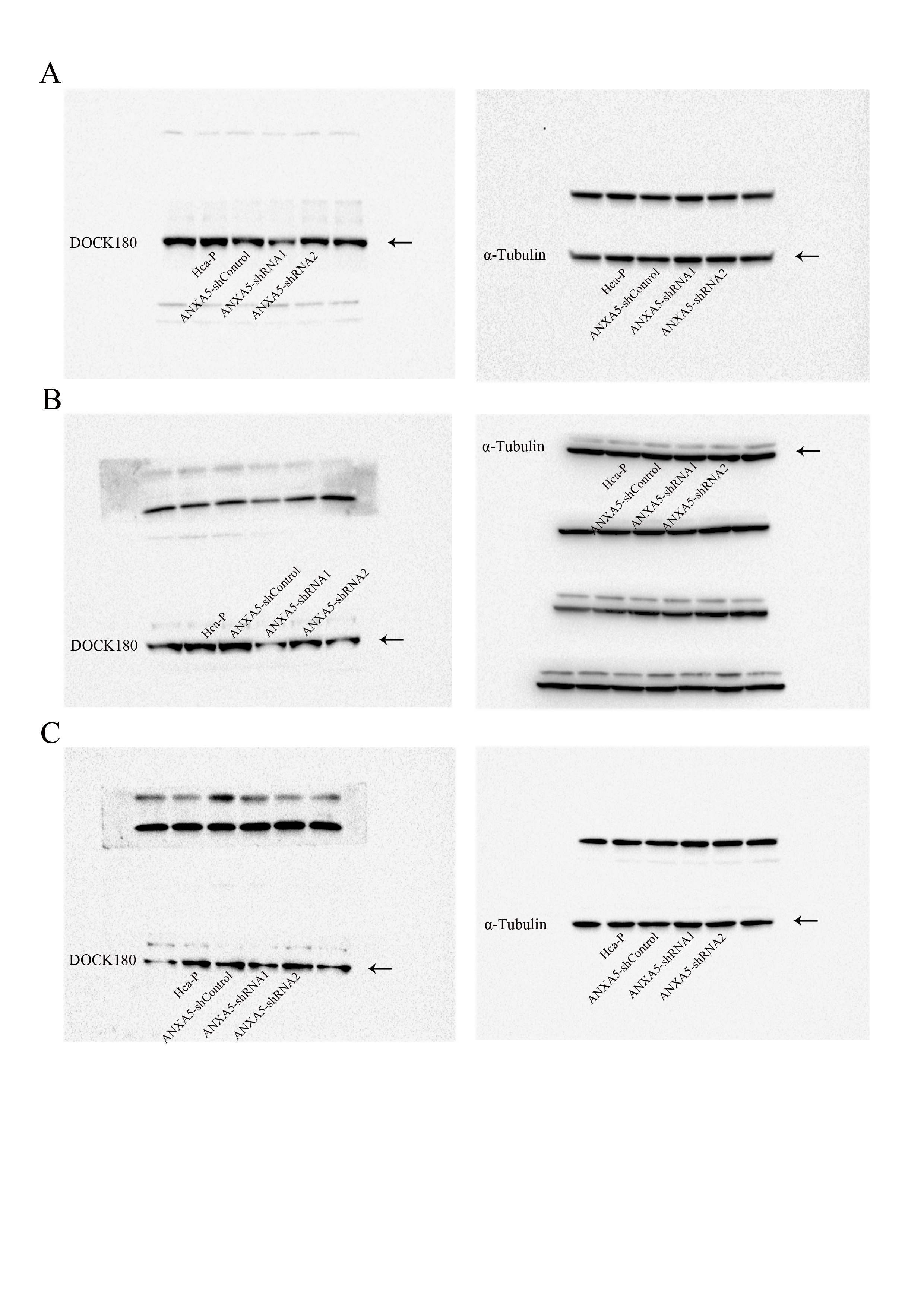


180

53

**Figure S2: The influence of ANXA5 stable knockdown on DOCK180 expression in Hca-P cells.** ANXA5 suppression reduced DOCK180 expression in Hca-P cells. (A) The first, (B) second and (C) third uncropped scans blots of DOCK180 and α-tubulin were from triplicate independent experiments. α-tubulin was used as the internal reference for relative quantification. The relative protein levels were quantified by analyzing the ratio of DOCK180 and α-tubulin grey intensity.


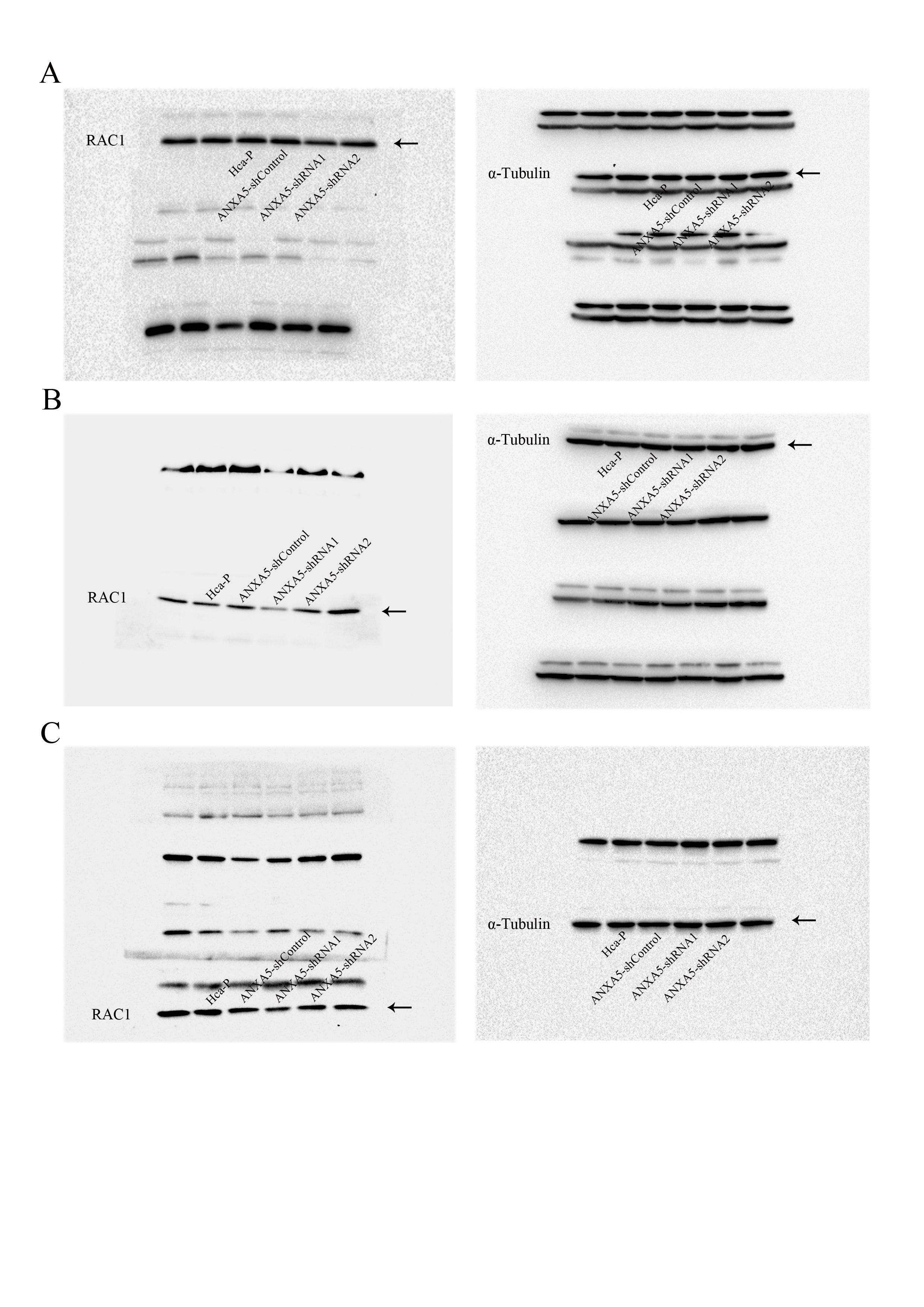


21

53

**Figure S3: The influence of ANXA5 downregulation on RAC1.** ANXA5 knockdown reduced the expression of RAC1 in Hca-P cells. (A) The first, (B) second and (C) third uncropped scans blots of RAC1 and internal reference protein α-tubulin were from triplicate independent experiments. The relative protein levels were quantified by analyzing the ratio of RAC1 and α-tubulin grey intensity.


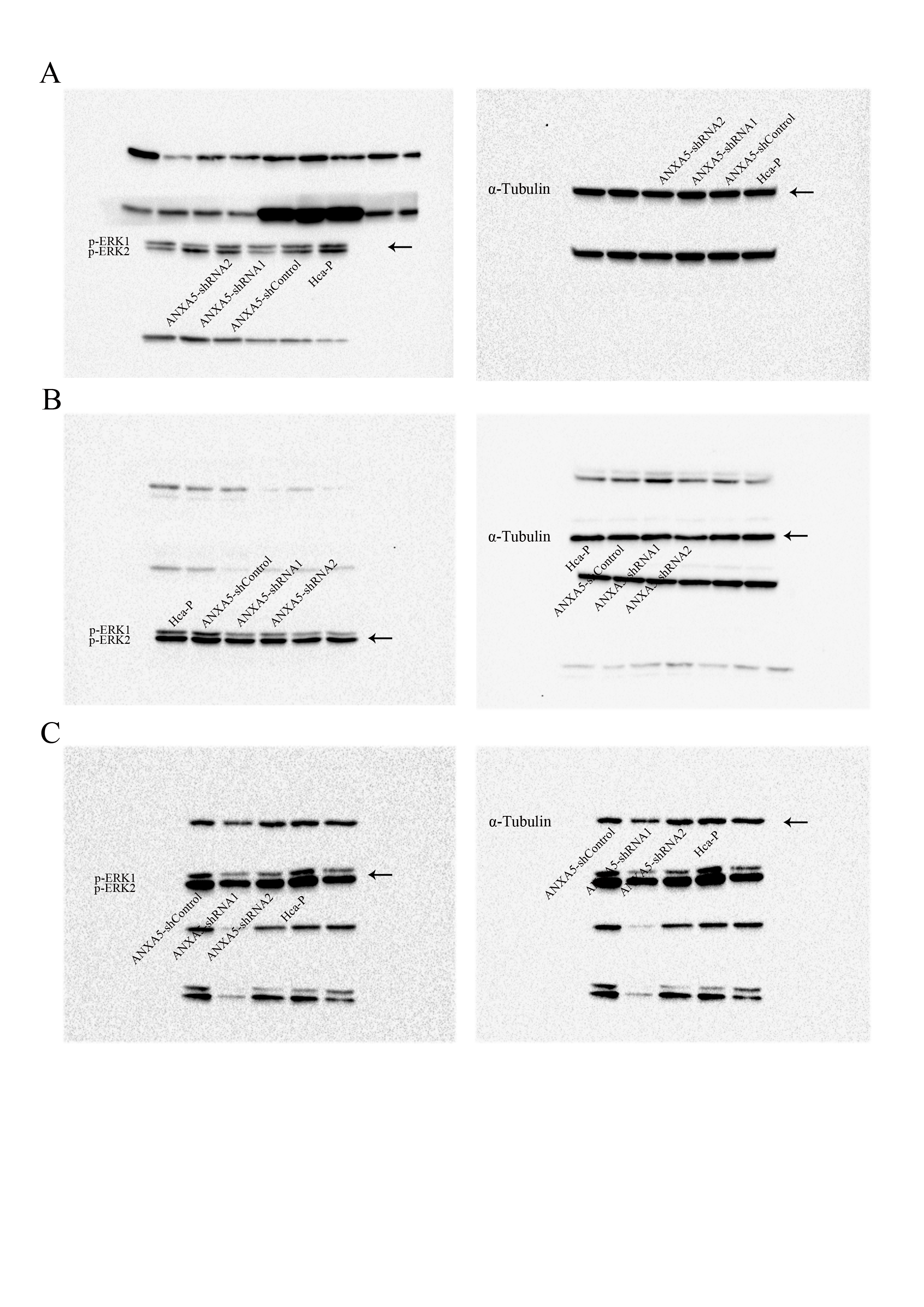


42

42

53

44

42

**Figure S4: ANXA5 knockdown reduced p-ERK1/2 expression in Hca-P.** (A) The first, (B) second and (C) third uncropped scans of p-ERK1/2 blots and internal reference protein α-tubulin of triplicate independent experiments. The relative protein expression levels were then quantified using the grey intensity of image band divided by the intensity of the internal standard α-tubulin.


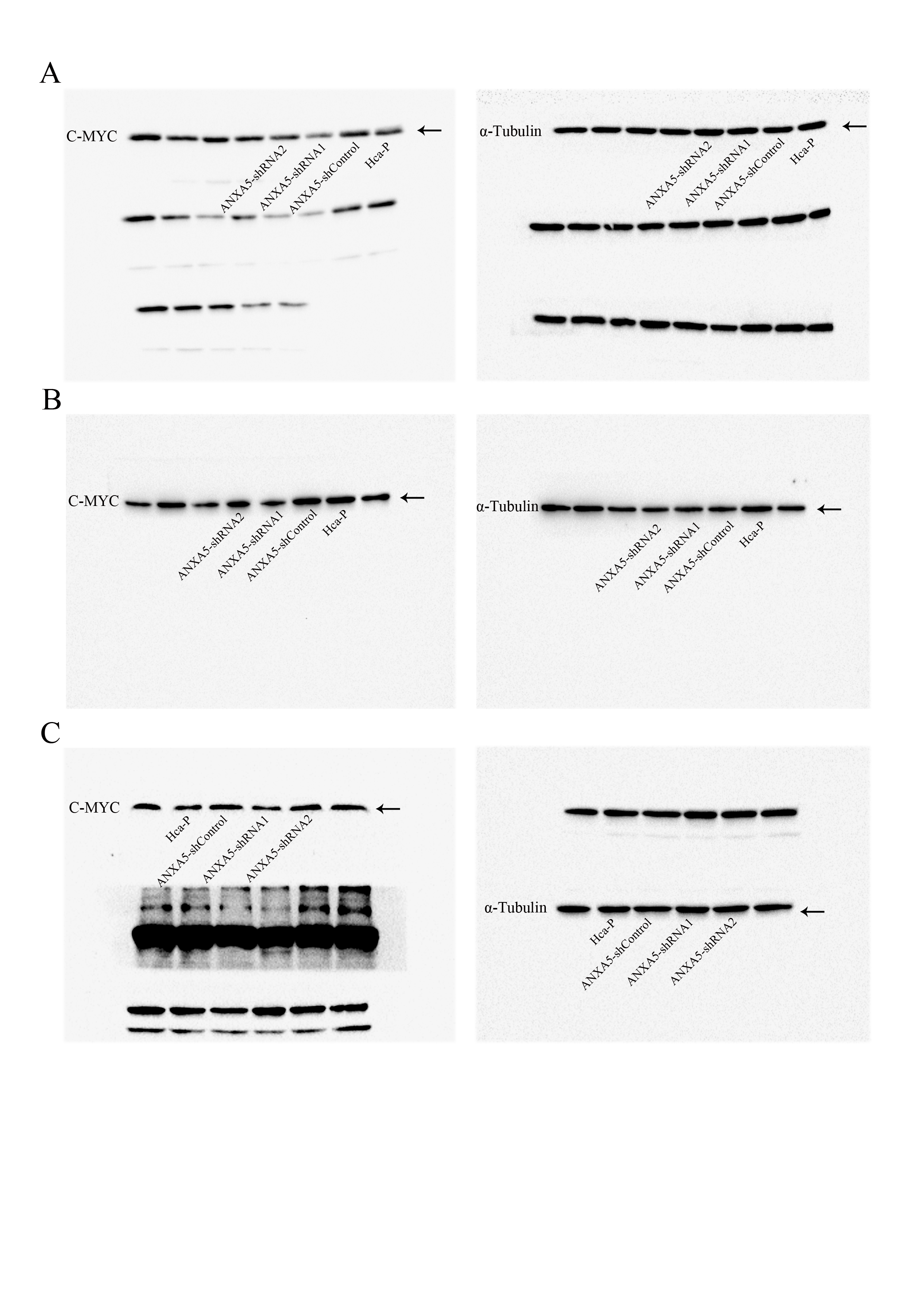


53

60

**Figure S5: ANXA5 suppression reduced C-MYC expression in Hca-P cells.** (A) The first, (B) second and (C) third uncropped scans of C-MYC blots and internal reference protein α-tubulin of triplicate independent experiments. The relative protein levels were then quantified using the grey intensity difference analysis method.
